# Prevalence of high-risk coronary plaques in patients with and without metabolic syndrome and the relationship with prognosis

**DOI:** 10.1186/s12872-020-01358-8

**Published:** 2020-02-11

**Authors:** Xu Yang, Wei Luo, Shan Han, Lei Zha, Jing Zhang, Xiaowei Li, Hui Zhao, Shuo Liang, Ru Zhao

**Affiliations:** 1 Department of Cardiology, Tianjin Chest Hospital, Tianjin, 300222 People’s Republic of China; 2 Department of Radiology, Tianjin Chest Hospital, Tianjin, 300222 People’s Republic of China

**Keywords:** Metabolic syndrome, Atherosclerosis, Risk factors, Prognosis

## Abstract

**Background:**

Metabolic syndrome (MS) is a disorder, characterized by clusters of cardiovascular risk factors such as central obesity, insulin resistance, dyslipidemia and hypertension. Patients with MS may have a higher plaque burden that increases their risk of major adverse cardiovascular events (MACEs). This study aimed to analyze the prevalence of high-risk coronary plaques in patients with and without MS by coronary computed tomography angiography (CCTA) and to investigate the relationship between MS, high-risk coronary plaques, and their prognosis.

**Methods:**

This was a retrospective cohort study of 1136 patients who underwent CCTA due to chest pain without obstructive heart disease (≥50% coronary stenosis) between January 2014 and December 2015 in our hospital. The relationships between high risk coronary plaques, MS, and other clinical factors were assessed. Multicollinearity analysis was performed to identify the collinearity between the variables. The proportional hazard assumption was checked and using Schoenfeld residual test. Cox proportional hazards model and Kaplan-Meier survival analysis assessed the relationship between MS, high-risk coronary plaques and MACEs.

**Results:**

High-risk plaques were more frequent in the MS group than non-MS group (*P* = 0.004). MS (HR = 2.128, 95%CI: 1.524–2.970, *P* < 0.001), presence of high-risk plaques (HR = 11.059, 95%CI: 7.749–57.232, *P* < 0.001) and high sensitivity C-reactive protein (hsCRP) (HR = 1.629, 95%CI: 1.128–2.352, *P* = 0.009) were related with an increased risk of MACEs in patients with risk factors for coronary heart disease. In patients with high-risk plaques, MS (HR = 2.265, 95%CI: 1.629–3.150, *P* < 0.001) and hsCRP (HR = 1.267, 95%CI: 1.191–1.348, *P* = 0.004) were related with an increased risk of MACEs. Kaplan-Meier analysis showed differences in MACEs between the MS and non-MS groups in the whole population and those with high-risk plaques (both *P* < 0.0001).

**Conclusions:**

High-risk plaques were more common in patients with MS. MS and the presence of high-risk plaques were independent risk factors for MACEs.

## Background

Metabolic syndrome (MS) occurs when an individual has a few risk factors, such as central obesity, insulin resistance, dyslipidemia and hypertension that together result in an increased risk of type 2 diabetes mellitus and cardiovascular disease [1]. In China, the prevalence of MS is around 33.9% overall with a prevalence of 31.0% in men and 36.8% in women [2]. As well as an increased risk of cardiovascular disease MS is also implicated in increased risk of sudden cardiac death [3]. Among the reasons for this is that factors associated with MS may increase coronary calcification and subclinical atherosclerosis (AS) [4].

AS may predict major adverse cardiac events (MACEs), a high-risk coronary plaque may progress rapidly and result in acute coronary syndrome [5]. Coronary computed tomography angiography (CCTA) can not only effectively evaluate the degree of coronary artery stenosis, but also analyze the coronary atherosclerotic plaque characteristics to identify high-risk plaques, which has unique advantages compared to other examinations [6]. CCTA studies used to mainly focus on distinguishing vessels by their degree of coronary stenosis. But in a histopathological study of ruptured plaques, 30% produced of nonobstructive ruptured plaques were further subdivided into those with luminal narrowing of 50–75% and those with luminal narrowing < 50% (25 and 5% of lesions respectively) [7]. Before the occurrence of acute coronary events, such patients often miss their optimal diagnosis time and treatment due to the lack of symptoms or the absence of severe stenosis of the coronary lumen. Therefore, detection of high-risk plaques may effectively reduce the occurrence of MACEs [8].

Identification of high-risk plaque features has been in the focus of coronary CTA imaging in the past years [9]. At present, positive remodeling, low computed tomography (CT) attenuation, napkin ring sign, and spotty calcification are all considered to be characteristics of high-risk plaques [6, 10], but the risk factors for rapid progression of high-risk plaques to MACEs are still unclear. MS is a complex disorder [11], and the factors involved aggregate inflammatory reactions in individuals and increases plaque instability [12]. A few studies have shown that patients with MS have a higher plaque burden and increase the risk of future occurrence of acute cardiovascular events and poor prognosis [10, 13–16].

The aim of the present study was to investigate the prevalence of high-risk coronary plaques in patients with and without MS and then to study the relationship between MS, high-risk plaques and prognosis.

## Methods

### Patients

This was a retrospective cohort study. Patients who underwent CCTA due to chest pain in our hospital from January 2014 to December 2015 were retrospectively analyzed. The inclusion criteria were: 1) Age ≥ 18 years old; 2) Patients who had chest pain and underwent CCTA; 3) Patients who had two or more risk factors of coronary heart disease (male > 45 years old, female > 55 years old; history of smoking; history of diabetes; high-density lipoprotein < 1.0 mmol/L; history of hypertension; family history of coronary heart disease occurred in < 55 years old males or < 65 years old women in first-degree relatives).

The exclusion criteria were as follows: 1) Patients who had a history of coronary revascularization or more than 50% stenosis of crosssectional luminal area shown by CCTA. 2) Patients who had arrhythmia, severe cardiac dysfunction (NYHA≥III degree); 3) Renal insufficiency (serum creatinine clearance rate, Scr > 120 ml/min); 4) Hyperthyroidism; 5) Contrast agent allergy history or the iodine allergy test was positive; 6) Poor quality of CCTA images.

This study was approved by the Institutional Review Board of our institution (the ethics number:2019LW-006). All patients underwent multidetector CT and had provided written informed consent after they agreed to participate in our study.

### Grouping

In accordance with the modified National Cholesterol Education Program–Adult Treatment Panel III criteria, an individual was defined as having MS if he or she had three or more of the following five criteria [17, 18]: (1) Waist circumference ≥ 90 cm in men and ≥ 80 cm in women using the International Obesity Task Force criteria for the Asian–Pacific population to determine waist circumference criteria; (2) Triglyceride levels ≥1.7 mmol/L; (3) HDL-cholesterol level < 0.9 mmol/L in men and < 1.0 mmol/L in women; (4) Blood pressure ≥ 130/85 mmHg or the use of antihypertensive medication; and (5) Fasting glucose level ≥ 6.1 mmol/L or the self-reported use of antidiabetic medication (insulin or oral agents). According to the above diagnostic criteria, the number of risk factors for metabolic syndrome in each patient was counted.

### CCTA examination

CCTA were conducted with dual source CT (SOMATOM DEFINITION, Siemens). Overlapping 64-layer images with a layer thickness of 0.6 mm were obtained. Before the CCTA examination, the process was explained to the patient so that they could cooperate with the examination during the scan. The scanning room was equipped with various rescue drugs, rescue equipment and oxygen. All patients underwent an iodine allergy test prior to the test. Patients who had heart rate > 70 beats/min were given an oral beta-blocker 30 min before CT coronary angiography.

During the examination, the patient’s anterior elbow vein was injected with a double-tube high-pressure syringe at a flow rate of 3.5–4.5 ml/s according to the patient’s weight. The contrast agent was Ultravist (370 mg I/100 ml, Bayer Health Care Co., Ltd) or Iohexol (350 mg I/100 ml, General Electric Pharmaceuticals). The scan range was 10–15 mm below the tracheal divergence to the palpebral surface (120 kV, 100 mAs) and the scan time was 6–10 s. Then, a coronary enhancement scan was performed, and the scanned images were processed and screened by multi-layer reconstruction (MPR), curved planar reconstruction (CPR), maximum intensity projection (MIP), volume rendering (VR) and cardiovascular optimization analysis software. Optimal CT images were used for the evaluation of coronary vascular plaques.

### CCTA image analysis

The coronary arteries were divided into 16 segments according to the American Heart Association classification method [19], and images were analyzed independently by 2 radiologists engaged in cardiovascular imaging diagnosis. When the results of the two doctors’ evaluations were inconsistent, they were read again and the opinions were unified. If their opinions were still not unified, another senior radiologist was engaged.

Coronary plaques were defined as having an area greater than 1 square millimeter visible at least in two mutually perpendicular orientations [20]. Obstructive coronary stenosis was defined as a coronary plaque causing luminal stenosis ≥50%. If CCTA did not show any coronary plaque (including calcification) the scan was defined as normal, if there was a plaque in any coronary artery with stenosis of < 50% this was defined as mild stenosis, if there was a plaque in any coronary artery resulting in 50–69% stenosis this was defined as moderately stenotic, a plaque resulting in stenosis of ≥70% was defined as severe stenosis [21, 22].

**Fig. 1:**
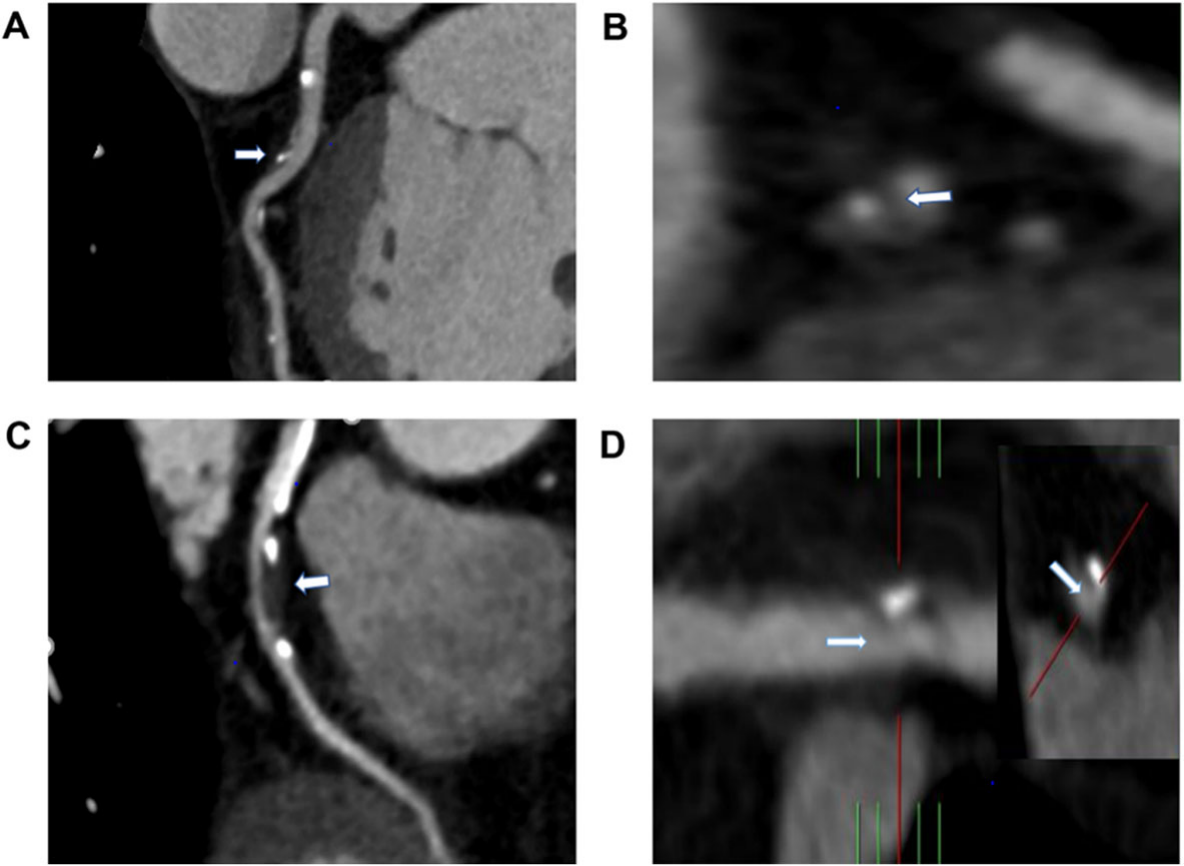
Characteristics of high-risk plaques (white arrow). **a** Spotty calcification; **b** Low attenuation; **c** Positive remodeling; **d** Napkin ring sign

As shown in Fig. 1, a high-risk plaque had at least one of the following characteristics [6, 20]: (1) Spotty calcification: defined as the presence of calcification with a diameter < 3 mm in the CCTA field, the length did not exceed 1.5 times the diameter of the lumen, and the width did not exceed 2/3 of the lumen diameter. (2) Low attenuation: plaque attenuation was assessed using a Hounsfield Unit (HU). If the average CT value of 3 interest areas randomly selected around the non-calcified plaque (the range is about 0.5–1.0mm^2^) was <30HU, the plaque was defined as low attenuation. (3) Positive remodeling: defined as the arterial remodeling index (RI = lesion plaque area/reference area) being greater than or equal to 1.1 (Reference segment: the same blood vessel, narrow and proximal 10 mm, where the lumen area was the largest, and the parts without major branches were defined as the proximal reference segment and the distal reference segment respectively). (4) Napkin ring sign: the central low attenuation lesion connected with the cavity was surrounded by a ring with slightly higher attenuation plaque tissue.

### Clinical data collection and follow-up

The general information of all subjects such as age, gender, body mass index (BMI), history of diabetes, hypertension, hyperlipidemia, blood pressure, fasting blood glucose (FBG), triglycerides (TG), low-density lipoprotein cholesterol (LDL), high-density lipoprotein cholesterol (HDL) and high sensitivity C-reactive protein (hsCRP) were collected. Furthermore, pharmacological treatment of hypertension, diabetes, hyperlipidemia and application of aspirin and statins were also recorded. During follow-up, a lifestyle of light diet, smoking cessation, alcohol restriction and moderate exercise is recommended.

Follow-up observation: the patients were followed up once every month for a period of 36 months. The pharmacological treatment of hypertension, diabetes, hyperlipidemia and application of aspirin and statins during the follow-up period were recorded unceasingly. The end point was the occurrence of MACEs including cardiac death, nonfatal myocardial infarction, and hospitalization for unstable angina or revascularization. Cardiac death was defined as death caused by acute myocardial infarction, ventricular arrhythmia, or refractory heart failure. Nonfatal myocardial infarction was defined as both ST-elevation myocardial infarction (STEMI) and unstable angina/non-STEMI (UA/NSTE-MI). Unstable angina was recorded as an event only in case urgent hospital admission was required.

### Statistical analysis

SPSS17.0 (SPSS Inc., Chicago, IL, USA) was used for all statistical analyses except proportional hazard assumption was checked by STATA 15.0 (StataCorp LLC StataCorp). Student’s t tests or Mann-Whitney U test were used for comparing continuous variables and χ2 tests for categorical variables. All data were expressed as the mean ± standard deviation (SD), median (interquartile range), or number (frequency). Possible multicollinearity was quantified using the variance infiltration factor (VIF). A VIF > 4.0 was considered an indication of harmful multicollinearity in the regression model. A Cox proportional hazard model was used to investigate any independent effect of MS, each individual MS component on the MACEs by adjusting significant variables. The proportional hazard assumption was checked using Schoenfeld residual tests. Kaplan-Meier survival analysis assessed the relationship between MS, high-risk coronary plaques and MACEs. All the tests were bilateral and *P* < 0.05 was considered statistically significant.

## Results

### Baseline characteristics

A total of 1136 patients were included, 488 were in the MS group and 648 in the non-MS group. Typical characteristics of high-risk plaques are shown in Fig. 1. There was no significant difference in age (*P* = 0.787) and gender (*P* = 0.857) between the two groups. Pharmacological treatment of hypertension (*P* = 0.518), diabetes (*P* = 0.079), hyperlipidemia (*P* = 0.201), aspirin (*P* = 0.147) and statins (*P* = 0.093) also showed no significant difference between the two groups. The BMI of the MS group was significantly higher than that of the non-MS group (26.6 ± 3.4 kg/cm^2^, 25.2 ± 3.3 kg/cm^2^, *P* = 0.001). Waist circumference was higher in the MS group compared to the non-MS group (93.7 ± 6.8 cm vs. 88.3 ± 6.3 cm, *P* < 0.001). There were higher rates of MACEs and high-risk plaques in the MS compared to the non-MS group (27.5% vs. 11.7%, *P* < 0.001; 66.8% vs. 59.3%, *P* = 0.004).(Table 1).

**Table 1:** Baseline clinical and biochemical characteristics in the MS group and non-MS group

	MS (*n* = 488)	Non-MS (*n* = 648)	P	VIF
Age (years), mean ± SD	61.5 ± 9.29	61.6 ± 8.70	0.787	1.056
Male, n (%)	240 (49.2)	323 (49.8)	0.857	1.125
BMI (kg/cm^2^), mean ± SD	26.6 ± 3.43	25.2 ± 3.31	0.001	2.820
Waist circumference (cm), mean ± SD	93.7 ± 6.80	88.3 ± 6.34	<0.001	2.780
Hypertension, n (%)	307 (62.9)	369 (56.9)	0.043	1.348
Diabetes, n (%)	306 (57.8)	425 (34.4)	<0.001	1.275
Hyperlipidemia, n (%)	276 (56.6)	261 (40.3)	<0.001	2.875
FBG (mmol/L), mean ± SD	6.5 ± 3.48	6.4 ± 3.66	0.046	1.071
LDL (mmol/l), mean ± SD	3.5 ± 0.76	3.2 ± 0.87	<0.001	2.725
TG (mmol/l), mean ± SD	1.8 ± 1.43	1.5 ± 0.92	0.001	1.070
HDL (mmol/l), mean ± SD	0.9 ± 0.69	1.0 ± 0.69	0.165	1.940
hsCRP (mg/l), mean ± SD	8.0 ± 2.72	7.1 ± 3.15	<0.001	1.417
Pharmacological treatment, n (%)				
Hypertension, n (%)	209 (42.9)	190 (44.8)	0.518	1.126
Diabetes, n (%)	187 (38.5)	217 (33.5)	0.079	1.193
Hyperlipidemia, n (%)	127 (26.1)	191 (29.5)	0.201	1.423
Aspirin, n (%)	223 (45.7)	268 (41.4)	0.147	1.106
Statins, n (%)	116 (23.8)	127 (19.6)	0.093	1.415
Presence of high-risk plaque, n (%)	326 (66.8)	378 (58.3)	0.004	2.998
MACEs, n (%)	144 (27.5)	76 (11.7)	<0.001	

*MS* metabolic syndrome; *BMI* body mass index; *FBG* fasting blood glucose; *LDL* low-density lipoprotein; *TG* triglycerides; *HDL* high-density lipoprotein; *hsCRP* high sensitivity C-reactive protein; *VIF* variance infiltration factor

### Characteristics of high-risk plaque analysis

Table 2 presents the data when only the 704 patients with high-risk plaques were considered (326 in the MS group and 378 in the non-MS group). There was no significant difference in age (*P* = 0.288) and gender (*P* = 0.577) between the two groups. Pharmacological treatment of hypertension (*P* = 0.501), diabetes(*P* = 0.069), hyperlipidemia(*P* = 0.132), aspirin (*P* = 0.112), and statins (*P* = 0.231) also showed no significant difference between the two groups. The hsCRP levels in the MS group were significantly higher than those in the non-MS group, and the differences were statistically significant (8.0 ± 2.7 mg/l vs. 7.1 ± 3.2 mg/l, P<0.001). In terms of the imaging characteristics of high-risk plaques in the two groups, the ratio of positive remodeling, spot-like calcification and napkin ring signs in the MS group were significantly higher than that in the non-MS group, and the difference was statistically significant (66.3% vs. 54.2%, *P* = 0.001; 65.3% vs 54.8%, *P* = 0.004; 50.6% vs. 40.7%, *P* = 0.010).

**Table 2:** Baseline clinical and biochemical characteristics in subjects with high risk plaques in the MS group and non-MS group

	MS with high-risk plaques (*n* = 326)	Non-MS with high-risk plaques (*n* = 378)	P	VIF
Age (years), mean ± SD	62 ± 10.4	61 ± 11.5	0.288	1.137
Male, n (%)	169 (52)	188 (50)	0.577	1.173
BMI (kg/cm^2^), mean ± SD	26.8 ± 3.67	24.7 ± 3.35	0.032	1.440
Waist circumference (cm), mean ± SD	91.8 ± 6.80	88.7 ± 6.34	0.026	1.348
Hypertension, n (%)	220 (67.5)	219 (58.0)	0.010	1.148
Diabetes, n (%)	217 (66.6)	207 (54.8)	0.002	1.138
Hyperlipidemia, n (%)	204 (62.6)	199 (52.6)	0.010	1.608
FBG (mmol/L), mean ± SD	6.7 ± 2.66	6.3 ± 2.27	0.006	1.125
LDL (mmol/l), mean ± SD	3.5 ± 0.78	3.4 ± 0.85	0.248	1.629
TG (mmol/l), mean ± SD	1.8 ± 1.16	1.6 ± 0.33	0.020	1.043
HDL (mmol/l), mean ± SD	1.2 ± 0.99	1.4 ± 0.74	0.043	1.080
hsCRP (mg/l), mean ± SD	8.0 ± 2.72	7.1 ± 3.15	<0.001	1.950
Pharmacological treatment, n (%)				
Hypertension, n (%)	141 (43.3)	154 (40.7)	0.501	1.144
Diabetes, n (%)	123 (37.7)	118 (31.3)	0.069	1.367
Hyperlipidemia, n (%)	81 (24.8)	76 (20.1)	0.132	1.021
Aspirin, n (%)	173 (53.1)	177 (46.8)	0.112	1.239
Statins, n(%)	94 (28.9)	93 (24.7)	0.231	1.069
Positive remodeling, n (%)	216 (66.3)	205 (54.2)	0.001	1.440
Low attenuation, n (%)	142 (43.6)	155 (41.0)	0.540	1.148
Spotty calcification, n (%)	213 (65.3)	207 (54.8)	0.004	1.264
Napkin ring sign, n (%)	165 (50.6)	154 (40.7)	0.010	2.203
MACEs, n (%)	96 (29)	57 (15)	<0.001	

*MS* metabolic syndrome; *BMI* body mass index; *FBG* fasting blood glucose; *LDL* low-density lipoprotein; *TG* triglycerides; *HDL* high-density lipoprotein; *hsCRP* high sensitivity C-reactive protein; *VIF* variance infiltration factor

### Follow-up

All the patients were followed up by telephone and outpatient service for 36 months. All Patients were encouraged to practice heart-healthy lifestyle behaviors [23], including: (1) Consume a dietary pattern that emphasizes intake of vegetables, fruits, and whole grains; includes low-fat dairy products, poultry, fish, legumes, nontropical vegetable oils, and nuts; (2) Limits intake of sodium, sweets, sugar-sweetened beverages, and red meats. (3) Counseling to reduce sodium intake by an average of 1150 mg/d. Engage in 2 h and 30 min per week of moderate-intensity physical activity. With a loss rate of 6.1%, 30 patients in the MS group and 48 patients in the non-MS group were lost to follow-up. Finally, 144 patients in the MS group had MACEs during the follow-up period (27.5%), while 76 patients in the non-MS group had MACEs during the follow-up period (11.7%), and the difference was statistically significant (*P* < 0.001). For those who had high risk plaques, 96 patients in the MS group had MACEs during the follow-up period (29%), while 57 patients in the non-MS group had MACEs during the follow-up period (15%), and the difference was statistically significant (*P* < 0.001). **(**Tables 1 and 2**).**

COX regression analysis was used to adjust for variables had statistical difference in Table 1, such as age, sex, BMI, waist circumference, LDL, TG, and FBG. The proportional hazard assumption was checked by the Schoenfeld residual global test, and there was no breach of that hypothesis (*P* = 0.159). Variance inflation factors were all < 4.0 indicated that there exist no significant interactions between the variables (Table 1). In the whole population, MS (HR = 2.128, 95%CI: 1.524–2.970, *P* < 0.001), presence of high-risk plaques (HR = 11.059, 95%CI: 7.749–57.232, *P* < 0.001) and hsCRP (HR = 1.629, 95%CI: 1.128–2.352, *P* = 0.009) were related to an increased risk of MACEs in patients with risk factors for coronary heart disease. Of the metabolic syndrome components, abdominal obesity (HR = 1.264, 95%CI:0.823–0.908, *P* = 0.033), hyperglycemia (HR = 1.567, 95%CI:1.096–2.639, *P* = 0.015), high blood pressure (HR = 1.700, 95%CI:0.297–0.728, *P* = 0.018) and hyperlipidemia (HR = 1.634, 95%CI:0.431–0.933, *P* = 0.021) were related to an increased risk of MACEs at 36 months. (Table 3).

**Table 3:** Multivariate COX regression analysis of risk factors for MACEs events in all patients (No. = 1136) at 36 months

	HR	95%CI	P
Metabolic syndrome, Yes vs. No	2.128	1.524–2.970	<0.001
Metabolic components			
Abdominal obesity, Yes vs. No	1.264	0.823–0.908	0.033
Hyperglycemia, Yes vs. No	1.567	1.096–2.639	0.015
High blood pressure, Yes vs. No	1.700	0.297–0.728	0.018
Hyperlipidemia, Yes vs. No	1.634	0.431–0.933	0.021
Presence of high-risk coronary plaque, Yes vs. No	11.059	7.749–57.232	<0.001
hsCRP, >10 mg/l VS ≤10 mg/L	1.629	1.128–2.352	0.009

Adjusted for age, sex, BMI, waist circumference, LDL, TG, FBG

**Fig. 2:**
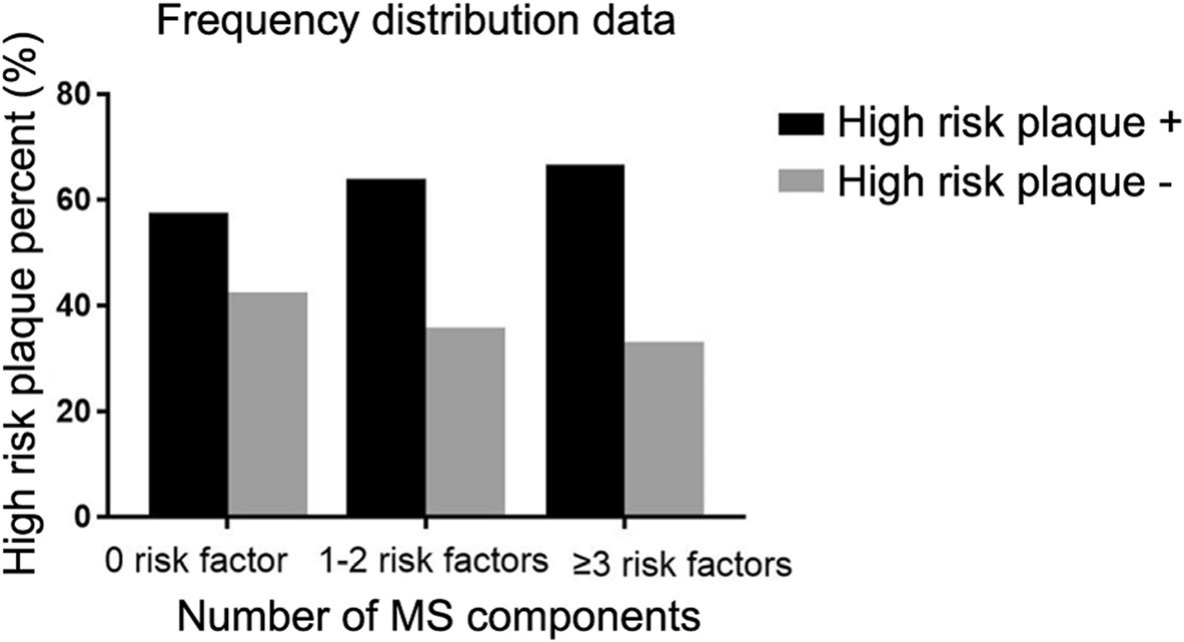
Graph showing the percentage of patients with and without high-risk plaques according to their number of metabolic syndrome components. The prevalence of high-risk coronary plaques increased as the number of metabolic syndrome components increased

Adjusted for statistically significant variables in Table 2 such as age, sex, BMI, waist circumference, HDL, TG, FBG, hsCRP, positive remodeling, spotty calcification, napkin ring sign, MS (HR = 2.265, 95%CI: 1.629–3.150, *P* < 0.001) and hsCRP (HR = 1.267, 95%CI: 1.191–1.348, *P* = 0.004) remained independent risk factors for MACEs in patients with high-risk coronary plaques at 36 months. Of the metabolic syndrome components, abdominal obesity (HR = 1.526, 95%CI:1.118–2.082, *P* = 0.008), hyperglycemia (HR = 1.640, 95%CI:0.460–0.890, *P* = 0.003) and high blood pressure (HR = 1.405, 95%CI:0.264–0.620, *P* < 0.001) were related to an increased risk of MACEs. (Table 4). We also tested the proportional hazard assumption by using Schoenfeld residual test and found no breach of that hypothesis (*P* = 0.116); Variance inflation factors were all < 4.0 so that there exist no significant interactions between the variables. **(**Table 2**)** Fig. 2 shows the number of patients with and without high-risk plaques according to the number of components of the metabolic syndrome they exhibited. As the number of MS components increased, the ratio of patients with high-risk plaques increased relative to the number without high-risk plaques.

**Table 4:** Multivariate COX regression analysis of risk factors for MACEs in patients with high risk plaques (No. = 704) at 36 months

	HR	95%CI	P
Metabolic syndrome, Yes vs. No	2.265	1.629–3.150	<0.001
Metabolic components			
Abdominal obesity, Yes vs. No	1.526	1.118–2.082	0.008
Hyperglycemia, Yes vs. No	1.640	0.460–0.890	0.003
High blood pressure, Yes vs. No	1.405	0.264–0.620	<0.001
Hyperlipidemia, Yes vs. No	1.277	0.898–1.817	0.174
hsCRP, >10 mg/l VS ≤10 mg/L	1.267	1.191–1.348	0.004

Adjusted for age, sex, BMI, waist circumference, HDL, TG, FBG, hsCRP, positive remodeling, spotty calcification, napkin ring sign

**Fig. 3:**
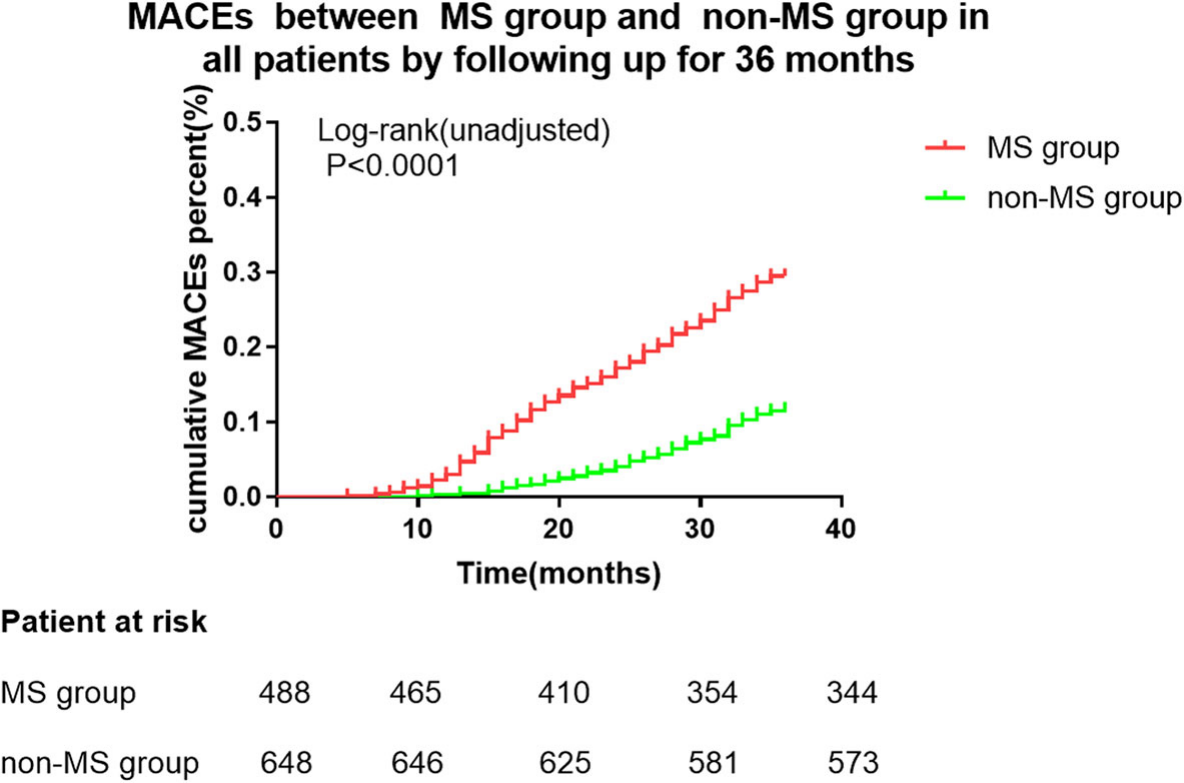
Kaplan-Meier survival curve showing MACEs in all patients followed up for 36 months

**Fig. 4:**
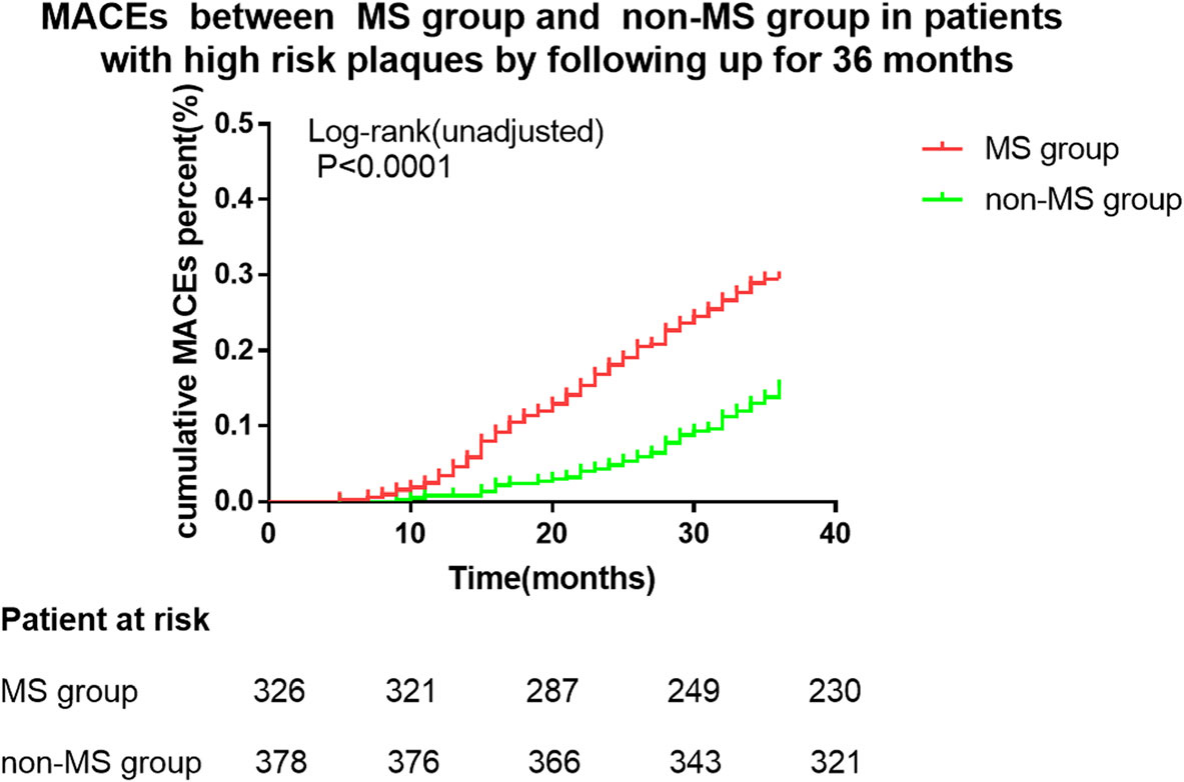
Kaplan-Meier survival curve showing MACEs of patients with high risk plaques followed up for 36 months

Kaplan-Meier survival curve analysis showed statistically significant differences in MACEs between the MS and non-MS groups in the whole population over 36 months (log rank *P* < 0.0001, Fig. 3). In the population with high-risk plaques there was also a significant difference in MACEs between the MS and non-MS groups over 36 months (log rank *P* < 0.0001, Fig. 4).

**Fig. 5:**
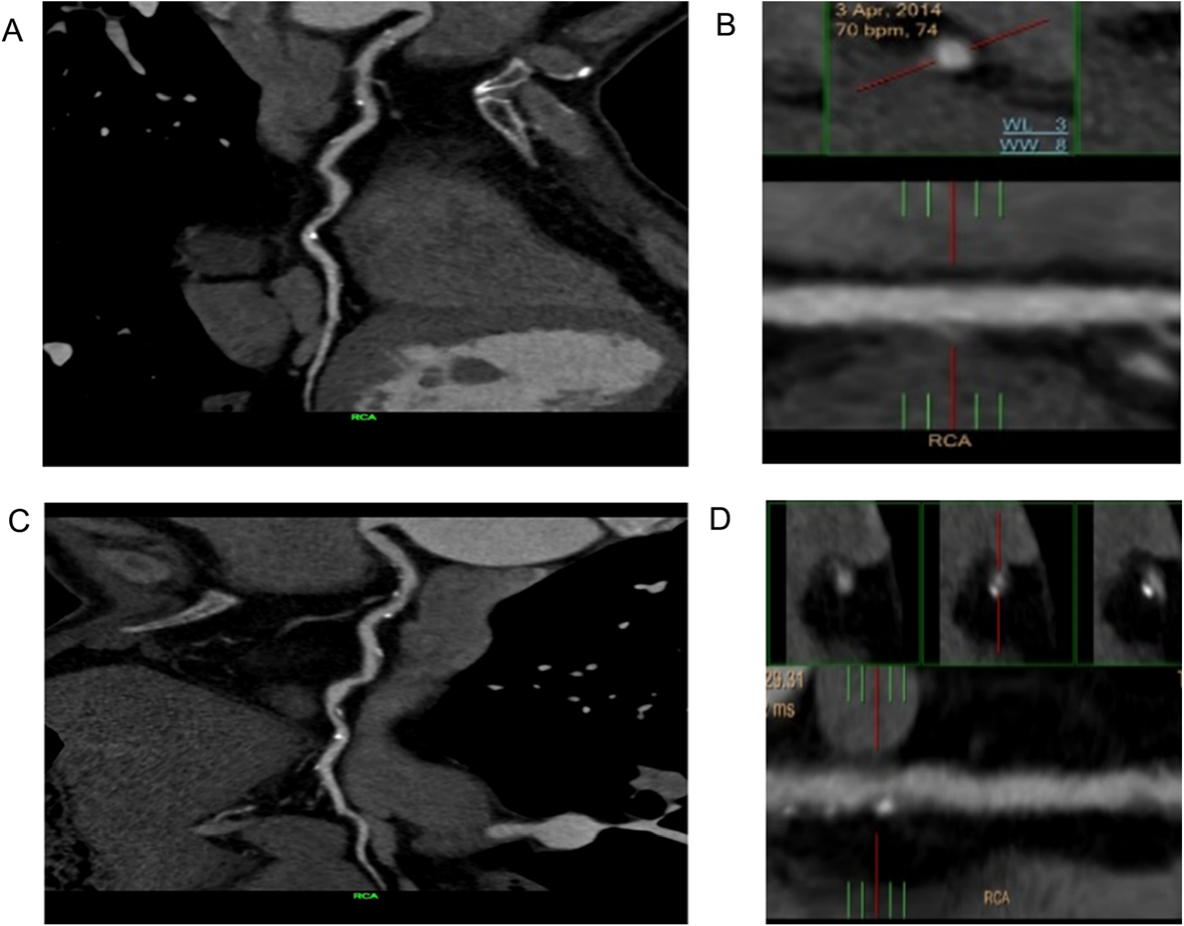
A 58-year-old male patient with metabolic syndrome. Mild stenosis was found in the right coronary artery by CCTA in April 2014, and the mixed plaque was recognized as high-risk plaque (positive remodeling, spotty calcification) (**a** - **b**). After 32 months, the patient underwent CCTA again due to unstable angina pectoris. The stenosis in right coronary artery developed into 80% in the proximal and 50% in the middle (**c** - **d**)

CCTA images from an example patient are presented in Fig. 5. The case was a 58-year-old male with metabolic syndrome, and the high-risk plaques (Fig. 5 a - b) developed into severe coronary stenosis (Fig. 5 c - d) in 32 months.

## Discussion

This study aimed to analyze the prevalence of high-risk coronary plaques in patients with and without MS by CCTA and to investigate the relationship between MS, high-risk coronary plaques, and MACEs. The results showed that there were more high-risk plaques and higher rates of MACEs in the MS group compared to the non-MS group. When the population with high-risk plaques was considered alone, MACEs remained higher in the MS group; hsCRP and the ratio of positive remodeling, spot-like calcification and napkin ring signs of the imaging characteristics of high-risk plaques in the MS group were significantly higher than that in non-MS group. COX regression analysis in the whole population showed metabolic syndrome, presence of a high-risk coronary plaque and abnormal hsCRP were risk factors for MACEs at 36 months. Of the metabolic syndrome components, abdominal obesity, hyperglycemia, high blood pressure and hyperlipidemia were all risk factors. Metabolic syndrome remained an independent risk factor for MACEs in patients with high-risk coronary plaques at 36 months and the other related risk factors were hsCRP, abdominal obesity, hyperglycemia, and high blood pressure. Kaplan-Meier survival curve analysis showed statistically significant differences in MACEs between the MS and non-MS groups in the whole population over 36 months and in the population with high-risk plaques.

Previous study has shown that metabolic syndrome has a relationship with atherosclerosis [4, 24]. In particular, the previous studies found the abdominal obesity and high blood pressure [4] or waist circumference and insulin resistance [25] components of metabolic syndrome were significantly associated with coronary plaques. In this study we found that the rate of high-risk plaques was higher in patients with metabolic syndrome. However, another study that used duplex ultrasound to investigate carotid plaque morphology in asymptomatic patients with and without metabolic syndrome found that metabolic syndrome did not affect the stenosis grade or the rate of unstable carotid plaques [25]. This study suggests that metabolic syndrome is more likely to lead to unstable plaques.

Insulin resistance is the central link of metabolic syndrome, and hsCRP concentration has been shown to be closely related to insulin resistance. It has been reported that serum hsCRP level of patients with cardiovascular disease combined with insulin resistance is higher than that of patients without insulin resistance, and it is positively correlated with saturated fatty acids and negatively correlated with unsaturated fatty acids [26]. Patients with high hsCRP level have more thin fibrous caps detected by optical correlation tomography (OCT), suggesting that hsCRP can reflect the instability of coronary plaque [27]. In this study, when the subpopulation of patients was investigated, the MS group had higher levels of hsCRP suggesting higher levels of inflammation and oxidative stress in patients with metabolic syndrome and providing a suitable pathophysiological environment for the development of high-risk plaques.

Based on characteristics such as low CT attenuation, napkin ring sign, positive remodeling and spotty calcification by CCTA, the high-risk plaques are vulnerable to rupture. The lesions with positive remodeling were larger in size and had more necrotic centers [28]. Spotty calcification tends to worsen the condition of patients with stable angina pectoris [29]. Napkin ring sign is the result of comparison between large necrotic centers (low central attenuation) and fibrous plaque tissues (slightly higher annular attenuation). In CT examination, napkin ring sign is used to identify severe coronary artery plaques and plaques covered with thin fibrous caps with high specificity, which can independently predict MACEs [30]. In patients with high-risk plaques in this study, the proportion of positive remodeling, spotty calcification and napkin ring sign in the MS group was significantly higher than that in the non-MS group, while there was no significant difference between the two groups with low CT attenuation, suggesting that metabolic syndrome has more influence on the size of high-risk plaques, the number of necrotic centers and the degree of calcification rather than the density of lipid in plaques. We are unsure why there is a difference between our results and the other study, but the patients with metabolic syndrome in the previous study used more classes of antihypertensive drugs and were treated with statins for a longer period than the patients without metabolic syndrome, so this may have caused some bias in the results. There are differences between the two studies in terms of the patient populations, because our study was in a Chinese population and the other study was undertaken in Brazil [25]. Also, there are some differences in the plaque analysis between these two studies. However, further investigation is needed to fully investigate the different conclusions of the studies. During follow-up the proportion of MACEs in patients with metabolic syndrome was significantly increased, and multiple regression analysis still indicated that metabolic syndrome was a risk factor for MACEs after adjustment for the relevant risk factors. This indicates that the calcification score of patients with metabolic syndrome progresses rapidly and is an independent risk factor for the progression of high-risk vulnerable coronary plaques [31]. Metabolic syndrome also increases the risk of coronary atherosclerosis in postmenopausal women through arterial stiffness [32]. In patients with metabolic syndrome, high-risk plaques are larger in size, have more thin fibrous caps and necrotic centers, and their higher levels of inflammation and oxidative stress can increase the instability of plaques. High-risk plaques are prone to rapid progress, rupture and induce coronary events. However, patients with metabolic syndrome themselves may have a higher plaque load and the number of high-risk plaques.

This study has some limitations. The study was quite small and from one hospital. A larger study would add more evidence for these results. As CCTA is not widely used in clinical screening for coronary heart disease in asymptomatic people, the relationship between high-risk plaques and metabolic syndrome in asymptomatic people was not included in this study, which may cause bias to the results. Besides, there is no evaluation of the cause of high-risk plaques. Whether metabolic syndrome is more likely to increase the vulnerability of a single plaque cannot be inferred, this requires further studies on dynamic change of single high-risk plaque.

## Conclusions

Comparison of patients with chest pain with and without metabolic syndrome showed that high-risk plaques were more common in patients with metabolic syndrome. Metabolic syndrome, in particular abdominal obesity, high blood pressure, and hyperlipidemia, and the presence of high-risk coronary plaques were all risk factors for MACEs. In patients with high-risk plaques MACEs related risk factors were hsCRP and metabolic syndrome, in particular abdominal obesity, hyperglycemia, and high blood pressure. Studies on the impact of metabolic syndrome on single high-risk plaque and the effect of every metabolic syndrome component on the prognosis of high-risk plaque are still needed. Subgroup analysis based on gender, age also should be discussed in future research.

## Data Availability

The datasets used and/or analysed during the current study are available from the corresponding author on reasonable request.

## Abbreviations

AS: Atherosclerosis
BMI: Body mass index
CCTA: Coronary computed tomography angiography
CPR: Curved planar reconstruction
FBG: Fasting blood glucose
HDL: High-density lipoprotein
hsCRP: High sensitivity C-reactive protein
HU: Hounsfield unit
LDL: Low-density lipoprotein
MACEs: Major adverse cardiovascular events
MIP: Maximum intensity projection
MPR: Multi-layer reconstruction
MS: Metabolic syndrome
SD: Standard deviation
STEMI: ST-elevation myocardial infarction
TG: Triglycerides
UA/NSTE-MI: Unstable angina/non-STEMI
VIF: Variance infiltration factor
VR: Volume rendering
